# Eccrine carcinoma : a rare cutaneous neoplasm

**DOI:** 10.1186/1746-1596-8-15

**Published:** 2013-02-04

**Authors:** Karima Idrissi Serhrouchni, Taoufiq Harmouch, Laila Chbani, Hind El Fatemi, Mohammed Sekal, Nawal Hammas, Meriem Soughi, Loubna Benchat, Afaf Amarti

**Affiliations:** 1 Department of Pathology, Hassan II University Hospital, Fez, 30000, Morocco; 2 Department of Dermatology, Hassan II University Hospital, Fez, 30000, Morocco

**Keywords:** Eccrine carcinoma, Sweat gland tumor, Mammary gland

## Abstract

Eccrine carcinoma is an extremely rare malignancy of the skin with few well documented cases reported in the literature. It is frequently found on the lower extremities, and it equally affects both sexes in the sixth and seventh decade.

In our case, we present a 46- year-old female with a recurring exophytic tumor on the right lower extremity, without local extension.

The initial tumor was biopsied, excised and diagnosed as an eccrine carcinoma.

**Virtual Slides:**

The virtual slide(s) for this article can be found here: http://www.diagnosticpathology.diagnomx.eu/vs/3568051328673318.

## Background

Malignant cutaneous adnexal neoplasms are a large and varied group, in particular eccrine carcinoma. They are one of the most challenging areas of Dermatopathology [1].

Eccrine and apocrine neoplasms present a bewildering array of morphologies which often defy precise classification [2].

The purpose of this case is to discuss the most common problems concerning the classification of this rare neoplasm and report the aim of the immunohistochemical profiles in differential diagnosis between a primitive eccrine carcinoma of the skin and a secondary neoplasm.

### Case report

**Figure 1:**
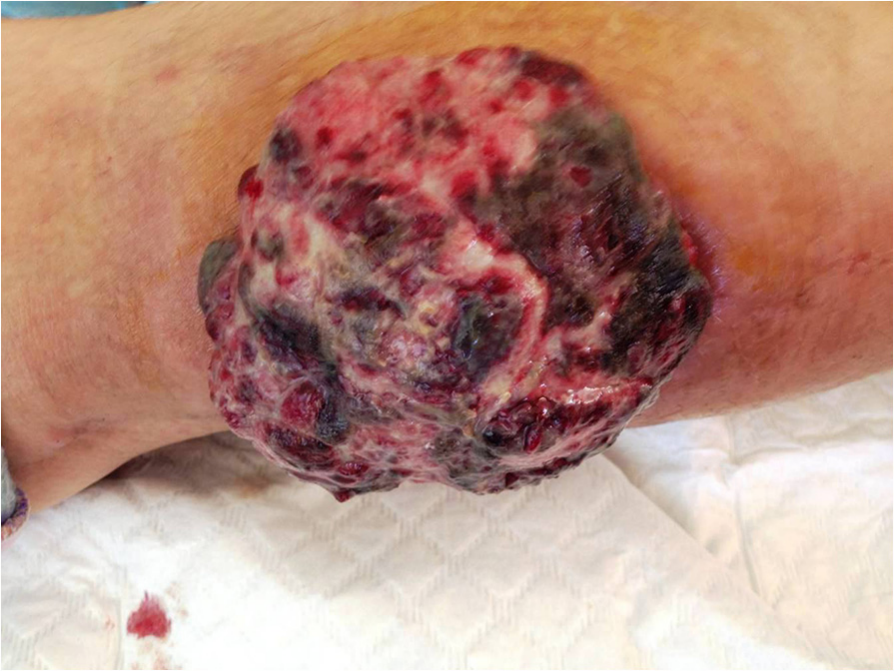
Clinical picture showed an ulcerative and bourgeoning mass of 15 cm with bleeding and purulent features on the posterior right lower extremity.

In July 2012, a 45-year-old Moroccan woman presented to the department of Dermatology of Hassan II University Hospital of Fez with a 25-year-history of primary infertility, and an 18-month-history of an exophytic mass at the posterior right lower extremity, gradually increasing in size. She was otherwise healthy and had no systemic symptoms. Physical examination showed an ulcerative and bourgeoning mass of 15 cm with bleeding and purulent features (Figure 1). Biological tests included a complete blood count, routine blood and urine chemistry were normal, except for elevated LDH. Tumor markers as like as CA 19–9, CA 125, CA 15–3 were normal. MRI of the leg showed a subcutaneous infiltrative process coming into contact with the muscle fascia.

The rest of the radiological examination, including radiography of the lung, abdominopelvic ultrasonography, computed tomographic thoraco-abdomino-pelvic scan, was normal.

The mammography and ultrasound examination objectified a 4 mm measuring benign cystic lesion of the left breast classified ACR 3, requiring supervision during six months.

After biopsy of the lesion, a surgical excision with clear margins was performed.

On gross examination, the tumor was 15 cm in size, bourgeoning, erythematous and heterogeneous with areas of necrosis and haemorrhage.

**Figure 2:**
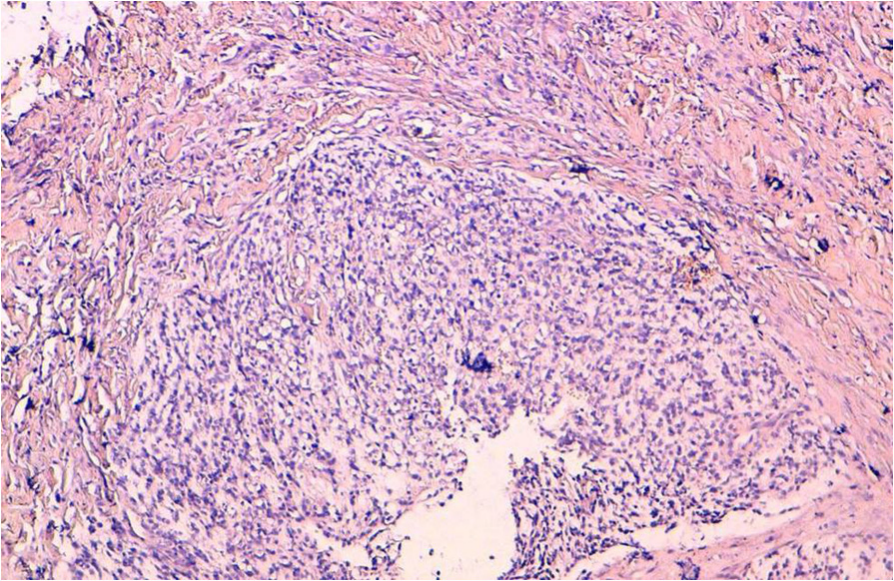
An undifferentiated malignant proliferation with nodular architecture, located in the deep dermis (hematoxylin-eosin, original magnification: x 100).

**Figure 3:**
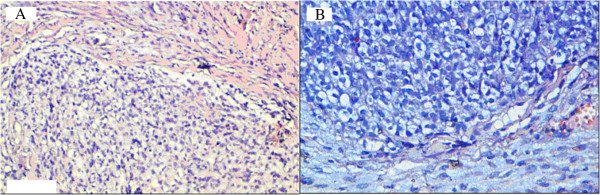
Tumor cells made clear cytoplasm with an hyperchromatic nucleus and a high mitotic activity (hematoxylin-eosin, original magnification: A, × 200, B, x 400).

Microscopic examination of the specimen revealed an undifferentiated malignant proliferation with nodular architecture, located in the deep dermis (Figure 2). Tumor cells made clear cytoplasm with an hyperchromatic nucleus and a high mitotic activity (Figure 3).

**Figure 4:**
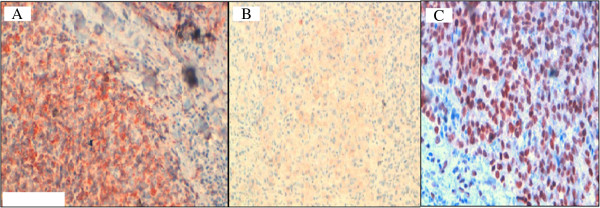
Immunohistochemical study showing reactivity for CK (A), CK7 (B) and progesterone receptors (C).

Immunohistochemical analysis showed positive staining for cytokeratine and progesterone receptor. Cytokeratine 7 was weakly positive and Ki67 was estimated at 95% (Figure 4).

In contrast, the tumor cells were not reactive to cytokeratine 20, estrogen receptor, LCA, CD10, S 100 protein, HMB45, desmin, H-caldesmon, CD 99, CD 117, CD 31 and CD 34.

Based on these data, the diagnosis of primary cutaneous eccrine carcinoma was retained.

## Discussion

Eccrine glands are directly developed from the embryonic epidermis in the early months of fetal development [1]. They are widely distributed almost everywhere on the skin [2]. The topographic distribution of adnexal structures also offers insight into the logical classification of adnexal neoplasms [3,4].

Microscopy alone is insufficient to establish eccrine lineage neoplasm because there are none specific microscopic features [3].

Some problems are related to the classification of sweat gland carcinomas, which are currently classified on the basis of the corresponding classification of benign sweat gland adenoma [5]. Such an approach, however, poses several problems; for example, (1) some carcinomas have no benign counterpart and do not fit the scheme (ductal carcinoma, adenoid cystic carcinoma, and mucinous carcinoma); (2) poorly differentiated carcinomas can be diagnosed only when a contiguous adenoma is found histologically; (3) histologic classification can be very complicated because adenomas are numerous and their classification is complex; and (4) terminology includes unusual and difficult terms, deriving from the terminology used for adenomas (malignant acrospiroma, porocarcinoma, hidradenocarcinoma, malignant cylindroma, malignant spiradenoma, and syringocystadenocarcinoma) [6-9].

Recently, a classification of sweat gland carcinomas designed based on the classification of breast carcinomas has been tentatively proposed [9].

Finally, recent studies have classified sweat gland carcinomas into eccrine and apocrine tumors [8,9]. In addition, no established authentic criteria are available for differentiation of an eccrine from an apocrine tumor [10]. Moreover, both eccrine and apocrine forms seem to exist in some categories [5,9,11,12].

The correct identification of the origin of tumor is of utmost importance for the determination of appropriate therapy and prognosis. Immunohistochemistry has proved to be a useful adjunct for this purpose [13].

The cells in the excretory coil of eccrine gland sweat express positivity for low molecular weight keratin, epithelial membrane antigen (EMA) and carcinoembryonic antigen (CEA), as well as S100 protein in the basal layer only. The myoepithelial cell layer may be highlighted by smooth muscle actin (SMA), p63 and calponin. Acrosyringeal cells stain for high molecular weight keratin and cytokeratin 14. A subpopulation of cells also expresses positivity to bcl-2 [2]. Some eccrine carcinomas are positif to estrogen and progesterone receptors wich has an important clinical implications, as affected patients may be partially treated with hormonal therapy [14]. The Ki-67 and p53 may be used to differentiate benign from malignant lesions [15].

Clinically, eccrine carcinoma must be considered in the differential diagnosis of patients older than fifty years with long standing tumors in the limbs, including basal cell carcinoma (BCC), Paget’s disease, melanoma, metastatic cancer [16], inflammatory, lymphocytic and vascular lesions [17].

The histopathology of mucinous eccrine carcinoma of the skin is analogous to that of its counterpart in the breast. Consequently, it is sometimes difficult to exclude a metastatic disease [15,18]. In a comparison between primary sweat gland tumors and metastatic breast carcinoma to skin, Busam et al. [13,19] found that the use of antibodies against epidermal growth factor receptor strongly decorated 81% of sweat gland tumors, but only 17% of metastatic breast cancers (P=0.001). There was no significant difference between the skin tumors and metastatic breast carcinoma when antibodies against estrogen and progesterone receptors were used, but with progression of disease, androgen receptors (AR) are preserved with higher frequency than ER/PR in metastatic mammary carcinoma [13]. In another study comparing benign, malignant primary eccrine and apocrine neoplasms to metastatic breast carcinoma, only 3.5% of these primary adnexal cancers demonstrated HER-2 positivity, whereas 10–23% of the breast carcinomas were positive [13,20]. The authors suggested that this test may be useful in distinguishing primary skin cancers from metastatic breast cancers [20]. Another study showed that primary cutaneous neoplasms stained strongly for p63 and CK 5/6 with high specificity, while CK 7 and 20 were not useful [15,21]. When CK 7 was positive in the cutaneous lesions, it exhibited marked focality and a specific pattern, while metastatic breast carcinoma expressed CK 7 diffusely and did not express p63 or CK 5/6. The usefulness of CK 5/6 was again shown by Plumb et al. [15,22]. Ivan et al. [15,23] presented further evidence of the usefulness of p63 in the diagnosis of adnexal cancer; in their study, none of the examples of metastatic adenocarcinoma to skin stained for p63, whereas virtually all the adnexal carcinomas were positive.

These studies showed that immunohistochemistry does not distinguish cutaneous eccrine tumours from cutaneous metastases of breast carcinoma, in which case clinical and radiological correlation is critical [24].

Other differential diagnoses comprise neoplasms with clear cell differentiation. These include trichilemmal carcinoma, clear cell BCC and clear cell carcinomas metastatic to the skin. With respect to the latter, the dominant diagnostic considerations are metastatic clear cell carcinoma from thyroid gland and lung. The expression of TTF seen in the great majority of these cases is helpful in resolving this dilemma. Some metastatic thyroid cancers also manifest expression of thyroglobulin. Metastatic clear cell carcinoma of renal primary origin manifests a prominent stromal vascularity with hemorrhage and areas of granular necrosis typically evident in biopsy material. Clear cell squamous cell carcinoma of the cervix is a glycogen-rich cancer with intercellular bridge formation characteristic of squamous differentiation [15].

## Conclusion

Eccrine carcinoma should be considered in the diagnosis of cutaneous malignant tumor with immunostaining for CK7, P63, CK5/6, estrogen and progesterone receptors.

## Consent from the patient

Written informed consent was obtained from patient for publication of this case report.

## Abbreviations

LDH: Lactate Dehydrogenase; MRI: Magnetic Resonance Imaging; ACR: American College of Radiology; CK: Cytokeratin; LCA: Leukocyte Common Antigen.

## Competing interests

The authors declare that they have no competing interests.

## Authors’ contributions

All authors read and approved the final manuscript.
